# Identification and quantification of projectile impact marks on bone: new experimental insights using osseous points

**DOI:** 10.1007/s12520-024-01944-3

**Published:** 2024-02-23

**Authors:** Reuven Yeshurun, Luc Doyon, José-Miguel Tejero, Rudolf Walter, Hannah Huber, Robin Andrews, Keiko Kitagawa

**Affiliations:** 1 Zinman Institute of Archaeology and School of Archaeology and Maritime Cultures, University of Haifa, Mt. Carmel, 3103301 Haifa, Israel; 2 UMR5199 PACEA, Université de Bordeaux, MCC, CNRS, 33615 Pessac CEDEX, France; 3 Seminari d’Estudis I Recerques Prehistòriques (SERP), University of Barcelona, Barcelona, Spain; 4 Department of Evolutionary Anthropology, University of Vienna, Vienna, Austria; 5 Human Evolution and Archeological Sciences (HEAS), University of Vienna, Vienna, Austria; 6 Early Prehistory and Quaternary Ecology, Department of Geosciences, University of Tübingen, Tübingen, Germany; 7 Ice Age Studio Hohle Fels, Schelklingen, Germany; 8 Early Prehistory and Quaternary Ecology, Department of Geosciences, University of Tübingen, Tübingen, Germany; 9 Senckenberg Centre for Human Evolution and Palaeoenvironment (SHEP), Tübingen, Germany

**Keywords:** Osseous points, Taphonomy, Zooarchaeology, Paleolithic hunting, Projectile impact marks (PIM), Experimental archaeology

## Abstract

Shifts in projectile technology potentially document human evolutionary milestones, such as adaptations for different environments and settlement dynamics. A relatively direct proxy for projectile technology is projectile impact marks (PIM) on archaeological bones. Increasing awareness and publication of experimental data sets have recently led to more identifications of PIM in various contexts, but diagnosing PIM from other types of bone-surface modifications, quantifying them, and inferring point size and material from the bone lesions need more substantiation. Here, we focus on PIM created by osseous projectiles, asking whether these could be effectively identified and separated from lithic-tipped weapons. We further discuss the basic question raised by recent PIM research in zooarchaeology: why PIM evidence is so rare in archaeofaunal assemblages (compared to other human-induced marks), even when they are explicitly sought. We present the experimental results of shooting two ungulate carcasses with bone and antler points, replicating those used in the early Upper Paleolithic of western Eurasia. Half of our hits resulted in PIM, confirming that this modification may have been originally abundant. However, we found that the probability of a skeletal element to be modified with PIM negatively correlates with its preservation potential, and that much of the produced bone damage would not be identifiable in a typical Paleolithic faunal assemblage. This quantification problem still leaves room for an insightful qualitative study of PIM. We complement previous research in presenting several diagnostic marks that retain preservation potential and may be used to suggest osseous, rather than lithic, projectile technology.

**Supplementary Information:**

The online version contains supplementary material available at 10.1007/s12520-024-01944-3.

## Introduction

The way Paleolithic hunters obtained their prey has been of enduring interest, as the direct traces are not normally visible in the archaeological record. Identifying hunting gear and hunting tactics informs us of the technology and behavior of particular human groups and may be extremely significant in documenting and explaining human evolutionary milestones. Humans adapting to new environments as a result of climate change or migration would need to adapt their weapons technology, which carries direct consequences to food procurement and therefore their fitness (Churchill 1993; Knecht 1997a, b; Shea and Sisk 2010; Lombard 2022); innovations in hunting gear would spread differently among foragers of varying degrees of connection and population structure (Tejero 2014; Doyon 2020).

The innovation and widespread use of composite weapons, usually in the form of a stone point attached to a haft in various configurations, is sporadically manifested in the lower Paleolithic/Early Stone Age (Wilkins et al. 2012) alongside rare finds of wooden weapons (Conard et al. 2020). Composite weapons became widespread in the archaeological record in the Middle Paleolithic of western Eurasia and the African Middle Stone Age (Knecht 1997a, b; Mithen 1999; Villa et al. 2009; Lazuén 2012; O’Driscoll and Thompson 2018; Lombard and Moncel 2023). Wooden spears have been in use through this period in northern Europe (Gaudzinski-Windheuser 2016), while the initial use of bow and arrow is suggested in Southern Africa (Bradfield et al. 2020) and Western Europe (Metz et al. 2023). Composite weapons further intensified in the Upper Paleolithic of western Eurasia, from ca. 45,000 years ago (Shea and Sisk 2010; Lombard 2022), with the proliferation of projectile technology that included spear throwers and darts that at times were composed of osseous points. Later, in the terminal Pleistocene and early Holocene, bow and arrow technology became more widely documented or inferred (Cattelain 1997; Yaroshevich et al. 2010). This description is very broad-brush, as innovations in hunting technology were often localized and stemmed from particular adaptations to local environmental shifts and human settlement dynamics, adding to existing technologies that had still been in use (O’Driscoll and Thompson 2018; Wood and Fitzhugh 2018; contra Ben-Dor and Barkai 2023).

Deciphering hunting methods, then, is important for understanding both the macro- and the micro-evolutionary processes of Paleolithic groups. Hunting methods may be deduced from the taxonomic composition of game (e.g., Wadley 2010; Yeshurun 2013), from the shape, size, manufacture techniques and damage patterns of the hunting implements (e.g., Knecht 1997a, b; Yaroshevich et al. 2010, Yaroshevich et al. 2023; Rots and Plisson 2014; Pétillon and Cattelain 2022; Lombard and Moncel 2023), or from the projectile impact marks (PIM) on the bones of the prey. The latter bears the most direct evidence for the relationship between the hunters’ technology and behavior, and the game individuals.

PIM occur when the projectile tip penetrates through the soft tissues of the animal with some force and becomes in contact with the bone. The shots can leave a range of marks that are dependent on a wide range of factors, including the weapon, the hunter’s action, and the target. Identifications of PIM have been reported in archaeological cases, both for human (e.g., Bocquentin and Bar-Yosef 2004; Churchill et al. 2009; Mirazon-Lahr et al. 2016; Chamel et al. 2017; Janković et al. 2017) and animal remains (Noe-Nygaard 1974, 1975; Bratlund 1991; Münzel and Conard 2004; Dewar et al. 2006; Leduc 2014; Yeshurun and Yaroshevich 2014; Duches et al. 2016, 2020; Pöllath et al. 2018; Gaudzinski-Windheuser et al. 2018; Wojtal et al. 2019). Nevertheless, the diagnosis, preservation potential, and variability of PIM are still not well-established, especially for organic-tipped weapons (for recent reviews, see O’Driscoll and Thompson 2014, 2018; Gaudzinski-Windheuser 2016; Pöllath et al. 2018).

PIM are extremely rare or absent in virtually all Paleolithic archaeofaunas, compared to other types of human-generated marks (butchery marks and percussion-induced fracture). The entire European Paleolithic record includes ~ 60 marked specimens (Smith et al. 2020). Even in some case studies where PIM were explicitly considered in the research design, very few or no PIM were discovered. Such was the case with the European Middle and Upper Paleolithic reindeer-dominated assemblages that were inspected by Castel (2008). The Eemian cervid assemblage of Neumark-Nord 1 (Germany) with exceptional preservation displayed PIM on two individuals out of 136, even though the authors conclude that the majority of the fauna derives from direct hunting by humans (Gaudzinski-Windheuser et al. 2018). The Middle Paleolithic fauna of Nesher Ramla (Israel) produced a single PIM (0.1% of NISP; Crater Gershtein et al. 2022; our ongoing work on a much larger sample failed to reveal additional PIM). The three Levantine Epipaleolithic (Natufian) faunal assemblages in Mount Carmel, Israel, that were inspected for PIM yielded between zero and two specimens bearing such modifications (0–0.02% of NISP; Yeshurun and Yaroshevich 2014). The Final Paleolithic and Mesolithic of Europe are thought to display more evidence of projectile weaponry, but in reality, the PIM abundance in their faunas is still very low. The marmot assemblage in Pradis Cave (Italy) produced 28 specimens with PIM, just 0.2% of NISP, despite the meticulous effort that was directed towards finding them, and the higher potential of small animals for being stigmatized with PIM (Duches et al. 2020). Even the Final Paleolithic Stellmoor assemblage (Germany), renowned for its relatively numerous evidence of PIM (which account for almost half of the entire European Paleolithic record: Smith et al. 2020), yielded just 26 specimens with embedded flint and an unspecified, but smaller, number of non-flint bearing PIM, out of ca. 18,000 specimens (~ 0.2%; Bratlund 1991).

The PIM paucity phenomenon still remains unresolved. This problem was noted before and some prevailing explanations were brought forward. Gaudzinski-Windheuser (2016) suggested that we overemphasize projectile technology, which, in reality, had not been used quite so often, at least before the Upper Paleolithic. Bratlund (1991) proposed that PIM are rarely produced by experienced hunters, and therefore would be rare to begin with. A prevailing explanation has been that PIM are particularly vulnerable to preservation processes and identification ambiguities (Smith et al. 2007; Castel 2008; O’Driscoll and Thompson 2014; Yeshurun and Yaroshevich 2014; Duches et al. 2016, 2020), but quantitative data to test this hypothesis are generally lacking.

Here we focus on osseous projectile implements, namely antler and bone points that are hafted distally and used with a spear thrower, manually, or with a bow and arrow. Osseous projectiles are a recent invention compared to the lithic projectiles and until now are mostly associated with anatomically and behaviorally modern humans (McBrearty and Brooks 2000; see, however, Julien et al. 2019). Their study is crucial in identifying the cultural and environmental adaptations of Upper Paleolithic populations (Tejero 2014; Langley et al. 2016; Tejero et al. 2016; Doyon 2020; Kitagawa and Conard 2020). Upper Paleolithic antler points, which in some regions become the most common osseous tools, have long been argued to be hunting implements (Knecht 1997a, b). Nevertheless, the direct link to the hunted game, through zooarchaeological and taphonomic analyses including osseous-induced PIM on animal bones, has been rarely demonstrated (but see Sinitsyn et al. 2019). Comprehensive experimental studies focused on the osseous points, launch mechanisms and fractures produced when armatures were launched into prey (e.g., Knecht 1997a, b; Pétillon 2006; Doyon and Katz Knecht 2014), and less on the corresponding traces on the bones (e.g., Stodiek 1993). Controlled experimental studies using this material are extremely rare, with a notable exception being the seminal study by Letourneux and Pétillon (2008). Their study entailed shots targeting carcasses 455 times using fork-based Magdalenian-type antler points which resulted in 127 impact traces on four different carcasses. They recorded notches, punctures, and perforations, and highlighted the more circular appearance of these traces when compared to experimental data from lithic-tipped projectile experiments. They also stressed the need for further experimental data to enable reliable comparison between the two tip types. Later, Gaudzinski-Windheuser and colleagues (2018) produced very similar circular damage by shooting a wooden spear, suggesting that lithic-tipped damage contrasts with “soft” tip damage, both osseous and wooden.

We present new experimental results, a detailed taphonomic assessment of the types and frequency of hunting lesions from shooting two animal targets with osseous projectiles. Our goal is to complement the bone damage reported by Letourneux and Pétillon (2008), and to clarify the identification of osseous-tipped vs. lithic-tipped weapons in light of the recently published PIM studies. Furthermore, we evaluate the PIM paucity problem by presenting the frequency of damages per skeletal element, analyzing the likelihood of receiving PIM per skeletal element, and considering the differential diagnosis of PIM in a complex taphonomic system.

## Materials and methods

### The shooting experiment

**Fig. 1:**
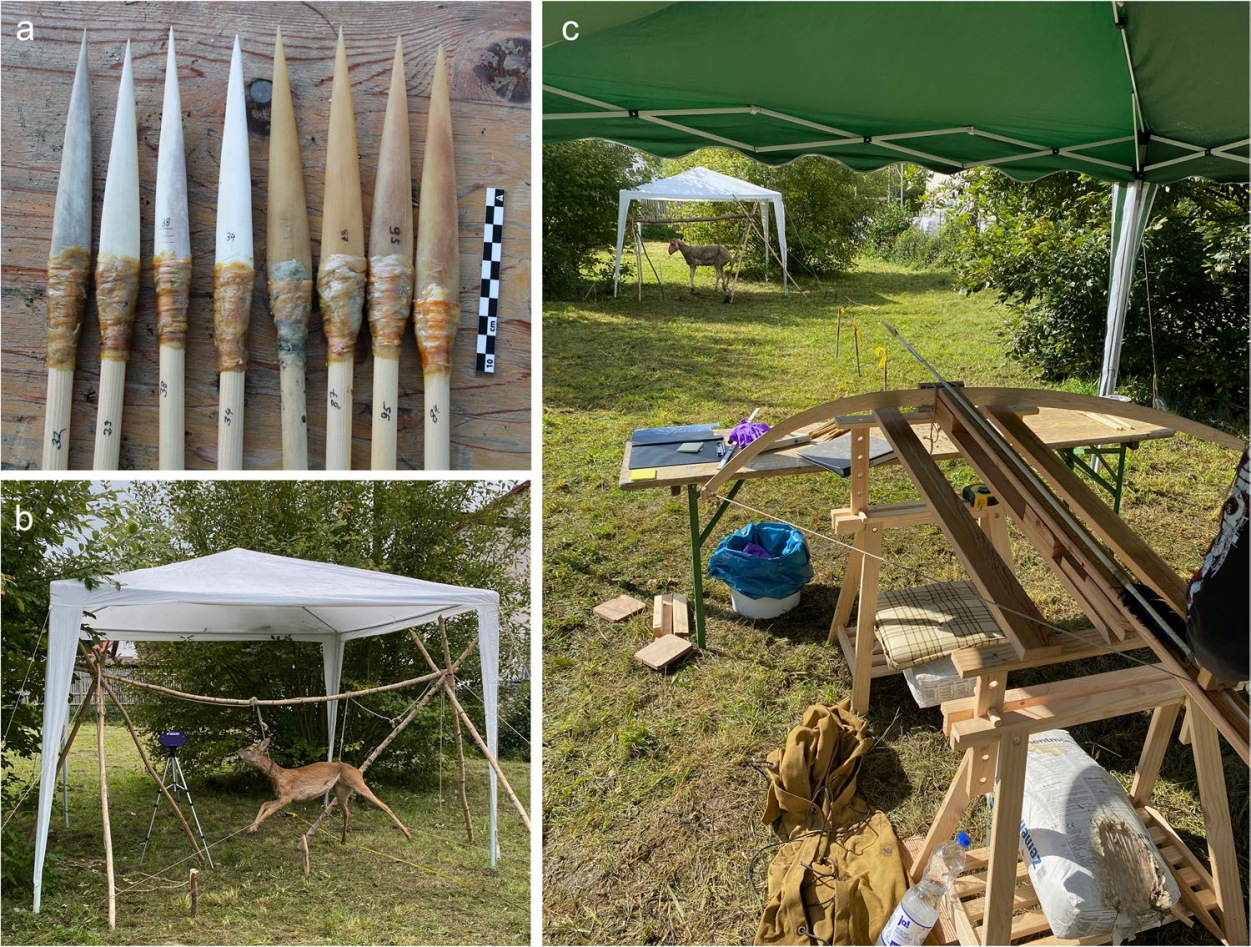
The shooting experiment: (**a**) selected bone and antler projectile replicas used in the shooting; (**b**) target 1 (sub-adult roe deer); (**c**) the calibrated crossbow and target 2 (adult sheep)

The shooting experiment was performed in September 2022 in Schelklingen, Germany. The targets were a 1-year old subadult female roe deer (*Capreolus capreolus*) as well as a 5-year old female sheep (*Ovis aries*), which was more robust than the former due to its larger size and age at death. The two target animals were purchased from a local hunter and a commercial butcher in compliance with relevant regulations. We experimented with four forms of osseous projectiles and with three different types of propelling mechanisms (Fig. 1). The projectile points were modeled after early Upper Paleolithic (Aurignacian) osseous points from Europe and Western Asia, including split-based points made of reindeer antler (small ones, mass 2.4 g on average, length 55 mm, width 12 mm, and large ones, mass 26.4 g on average, length 173 mm, width 24 mm), and massive-based points made of horse metapodial bones that were untreated but stored for several months in a cool and dry place (small ones, mass 8.1 g on average, length 103 mm, width 12 mm, and large ones, mass 35.7 g on average, length 165–179 mm, width 24–26 mm).

We performed 110 shots of 26 points from a distance of 13 m at two targets with 65 hits. Different modes of shot include spear-throwing by hand and a spearthrower, handmade crossbow machine and bow and arrow. The average speed of the shots measured 21.1 mps, with the range of 12 and 40 mps. The osseous-tipped projectiles proved to be quite effective, penetrating on average 16 cm (*n* = 57, *sd* = 10.79, range: 1–57 cm) into the unprocessed carcasses. Following each hit, we recorded the precise anatomical location of the hit and the bone/s that came into contact with the projectile by a manual examination of the full depth of penetration. In this study, it was not possible to identify individual shots that corresponded to the damages, because the projectiles landed at times on the same locations of the animal and most aims were not randomized for the purpose of this study. The speed of the shots was slower on average for the crossbow machine than those that were shot manually.

### Bone analysis procedure

The preparation of the bones entailed suspending the animals from their rear limbs and cutting off the extremities, and therefore is unlikely to inflict any cracking or breakage on the limb bones and axial elements other than the metapodials. Thereafter, the skeletal elements were brought to a maceration laboratory where soft tissues were removed using a metal surgical knife and the bones were macerated around 50 °C water with dish soap for a few days. We assumed that all modifications, except for the metal knife cutmarks with V-shape striation marks, were the result of the shooting. These cutmarks were noted but excluded from our analysis.

We classified all bone modifications according to the categories suggested by Letourneux and Pétillon’s (2008) scheme, which was based on Morel’s (1993) classification. Our categories only deviate from those of Letourneux and Pétillon’s (2008) by making no distinction between “primary” and “secondary” marks and an addition of a sixth category. We used their classification system because of its straightforwardness, inclusiveness, and convenience of use in describing experimental and archaeological PIM (e.g., Letourneux and Pétillon 2008; Yeshurun and Yaroshevich 2014). The categories are not mutually exclusive; a PIM could consist of two or more categories. We searched for: (1) *Notches*: a removal of a small amount of material from the edge of the bone; (2) *Punctures*: damage or shallow indentation going within the bone that results from the contact with the projectile tips; (3) *Perforations*: damage resulting from the projectiles that pierce through the bone and leave a hole; (4) *Embedding*: the point, or parts thereof, remaining lodged within the bone after the puncture or perforation; (5) *Cracking*: cracks that spread from the impact point, sometimes causing the entire bone to split or fragment. We added an additional category, which is considered in flint PIM studies (e.g., Smith et al. 2007): (6) *Striations*: linear lesions caused by the point dragging along the bone, usually perpendicular to its longitudinal axis; they do not involve flaking the bone or tearing off much bone material as in notches. We note that in the classification proposed by O’Driscoll and Thompsons (2014), striations seem to be lumped with notches in the “drag marks” category and punctures with perforations. Such lumping would hinder our descriptions for accuracy and hence we decided to follow Letourneux and Pétillon (2008).

All the bones of the two target animals except for the head and limb extremities (the carpals, tarsals, metapodials, and phalanges) were systematically examined, employing a typical method that we employ for zooarchaeological analysis. We examined all bones with a stereoscopic microscope (Zeiss Discovery.V12) with a high-intensity oblique light source, at 8–40 × magnification, following the procedure outlined in Blumenschine et al. (1996) to systematically detect and classify bone surface modifications. Two experienced analysts (K.K. and R.Y.) examined each specimen and cross-checked their classifications. Since the bones from the experiment were not subjected to any taphonomic process other than shooting with osseous-tipped weapons and butchery with metal knives, all marks were considered to belong to either of these agents. The metal knife cutmarks were observed on 29 bones, appearing as very thin, short striations on all anatomical parts. The ensuing description pertains to the PIM modifications only.

## Results

We performed 110 shots with antler and bone-tipped projectiles and hit the targets 65 times, out of these eight hits were to the stomach and could not have hit any bone. The remaining 57 hits produced 29 bone damages on 22 bones (Table 1), i.e., a visible bone modification occurred on average in every other hit. The majority of bones (15 of 22, 68%) exhibited one kind of modification, while the rest exhibited a combination of modifications either resulting from multiple shots or single shots that caused multiple forms of modification; the latter were counted as a single PIM (Table 1). All marked specimens are available to view in 3D as downloadable pdf files (Supplementary online data).

**Table 1:** Description of the bone specimens modified by our shooting. The two targets are roe deer (*Capreolus capreolus*) and domestic sheep (*Ovis aries*)

#	Animal	Side	Bone	Description
3	*Capreolus*	R	Rib	**Cracking** on mesial (jagged) and lateral (a major wide crack with an undetached flake). The cracked area is adjacent to the distal end; its center is ca. 30 mm from the distal end
5	*Capreolus*	R	Rib	**Perforation,** 34 mm from the distal end, 5 mm in diameter, causing a longitudinal **crack** of 60-mm long and other smaller cracks running obliquely. The perforation caused a slight offset, depressed laterally (the projectile hit from the left side)
22	*Capreolus*	L	Rib	**Cracking** and detachment of the two halves of the bone, caused by a hit approximately in the middle of the rib, in the thin part of the shaft
25 (A)	*Capreolus*	L	Scapula	**Perforation** on the thin caudal blade, measuring 25 × 10 with internal beveling present
25 (B)	*Capreolus*	L	Scapula	**Perforation** on the thin caudal blade, measuring 20 × 10 with internal beveling present. Some associated **cracking**
26	*Capreolus*	R	Scapula	**Perforation** 6 × 4 mm wide from a hit just by the spine, in the thin portion, that passed through the entire breadth of the animal, leaving a small oval hole on the mesial side and an offset on the lateral side
38	*Capreolus*		Cervical vert	A wide **notch** on the thin left side wing, ventral aspect, 15 mm in diameter. Slight internal beveling is seen on the exit side
45	*Capreolus*	R	Rib	**Cracking** on the mid-shaft, roughly equal between the two rib extremities. The cracking is seen on both aspects, but more on the ventral side. Flaking occurred but is still undetached; slight offset visible externally (the strike came from the left)
51	*Capreolus*		Thoracic vert	A **notch** showing the direction of the hit (from left to right) and a detached dorsal process, likely due to **cracking** as a result of the hit
64 (A)	*Capreolus*	R	Ulna	A wide **striation,** perpendicularly oriented, on the medial aspect, posterior to the olecranon
64 (B)	*Capreolus*	R	Ulna	Removal of the proximal end, likely from a hit that caused **cracking** of this area
76	*Ovis*	R	Rib	A **striation** oriented obliquely, about 80 mm from the rib head, showing slightly curved trajectory and a relatively V-shaped cross-section
81 (A)	*Ovis*	L	Rib	A perpendicularly-oriented **striation**, a lesion on the mesial aspect, in equal distance from the two extremities of the rib
81 (B)	*Ovis*	L	Rib	A perpendicularly-oriented **striation**, a wider lesion across the entire height of the rib, with u-shaped cross-section, 40 mm from the rib head
83 (A)	*Ovis*	L	Rib	A **perforation** (semi-circular), 40 mm away from the distal end in a diameter of 20 mm. The entry was lateral and the mesial side shows internal beveling
83 (B)	*Ovis*	L	Rib	A **notch**, located 80 mm from the distal end, 25-mm wide with an offset slight offset mesially
90	*Ovis*	L	Rib	A **notch** located less than 1 cm from the distal edge (maximum diameter, 30 mm). The notch shows lateral entry and internal beveling on mesial side
92	*Ovis*	L	Rib	The proximal part of the rib snapped off, likely from a hit that caused **cracking** and the detachment of the rib head
93 (A)	*Ovis*	L	Rib	**Notch**, located 5 cm from the distal end (maximum diameter, 24 mm), showing entry in posterior position and offset internally. A bone chip was observed within the notch, which could either be at the same bone or from the antler point
93 (B)	*Ovis*	L	Rib	**Notch**, located at the distal end, at a thicker portion of the rib, about 20 mm in diameter with a narrower indentation, about 5-mm wide. No visible beveling
96	*Ovis*	L	Rib	**Perforation** that caused widespread cracking on the distal shaft of the rib. The hit was 35 mm from the distal end, causing a crack extending 80 mm longitudinally, and across the entire height of the rib. Some offset is visible mesially
98 (A)	*Ovis*	L	Scapula	**Perforation** on the lateral-caudal surface at a diameter of 17 × 10 mm, showing lateral entry and an offset (no beveling) on the mesial side
98 (B)	*Ovis*	L	Scapula	**Notch** on the scapula neck, a surviving indentation measuring 8 mm on the caudal-lateral edge, which caused cracking and breakage of this part
123 (A)	*Ovis*		Lumbar vert	**Perforation** on the dorsal spine 10 × 6 mm, left entry, offset on right, cracked the entire dorsal process leaving some bone chips still undetached
123 (B)	*Ovis*		Lumbar vert	**Notch** on the left-caudal body, under the inferior articular process, and corresponding **perforation** on the right side, in oblique angle, at the base of right lateral process. The peroration is ca. 2 mm in diameter
136	*Ovis*	L	Ulna	A subtle **notch** on the caudal aspect of the proximal ulna, 15-mm wide, showing a small detachment of a flake
13 + 24	*Capreolus*	L	Rib	**Cracking** and detachment of the two halves of the bone, caused by a hit approximately in the middle of the rib, in the thin part of the shaft
15 + 55	*Capreolus*	R	Rib	**Cracking** and detachment of the two halves of the bone, caused by a hit approximately in the middle of the rib, in the thin part of the shaft
56 + 21	*Capreolus*		Rib	**Perforation,** about mid-shaft, 35 mm from the distal end, causing longitudinal **cracking** minimally 50-mm long and causing the breakage from the other part of the rib. Slight offset visible

In all, we identified four types of PIM, sometimes in combination with each other (Table 1): perforations, cracking, notches, and striations. No punctures (i.e., incomplete breaching of the bone) and embedded tip fragments were identified. Regarding the last point, we did observe at least one bone chip within a notch (#93-A), but we could not ascertain whether it dislodged from the struck target or the osseous projectile. We also shot a single ivory point that got embedded in the bone, but as it was a pilot, it was excluded from this study that focused on the quantitative analysis of bone and antler points.

**Fig. 2:**
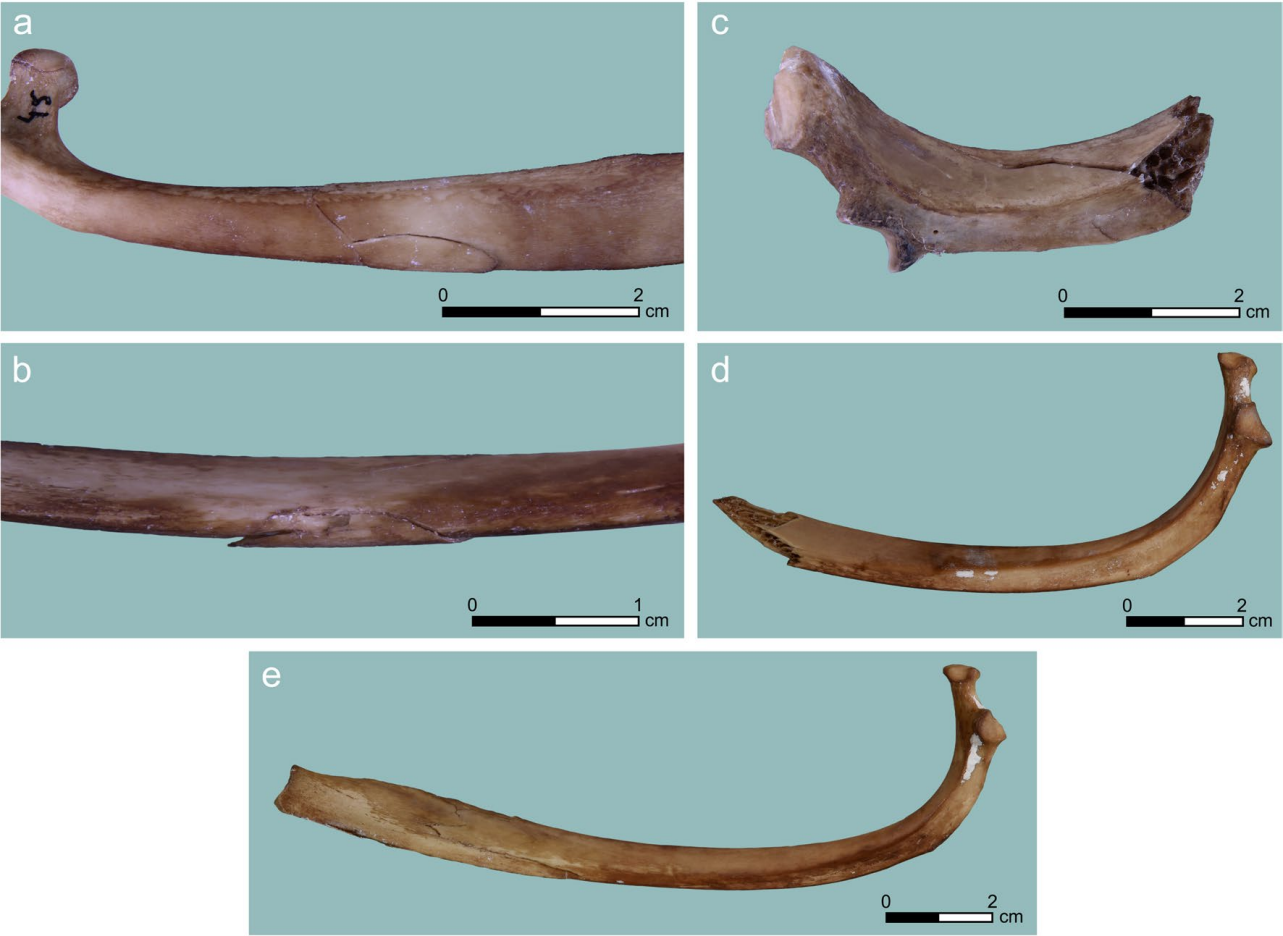
Cracking PIM generated in our experiment: (**a**, **b**) ventral and dorsal views on a cracked rib (#45) with an undetached flake; (**c**) a rib (#92) showing deep cracks and complete bone splitting; (**d**) a rib that was hit on the thin part of the shaft, causing cracking and splitting of the bone (#15 + 55); (**e**) cracking on mesial aspect of a rib (#3)

*Cracking* constituted the exclusive PIM damage in seven cases and also accompanied perforation and notch damage in four and one cases, respectively. It was found exclusively on flat bones: the ribs, a proximal ulna, a scapula blade, and a thoracic vertebra process (Table 2). This damage type is variable, ranging from shallow but extensive cracks (up to 80-mm long), to deep ones that resulted in rib splitting or near-detachment of bone flakes (Fig. 2). When cracks were the sole feature (without other PIM type), their morphological traits could not be used to distinguish them archaeologically from other bone-modification processes that can cause cracking pre- and post-depositionally.

**Table 2:** Counts of studied bones from the two target animals and PIM types per bone element

	Unmodified	Cracking	Striation	Notch	Notch and cracking	Perforation	Perforation and cracking	Perforation and notch	Total
Atlas	1								1
Axis	2								2
Cervical vert	8			1					9
Femur	4								4
Humerus	4								4
Innominate	3								3
Lumbar vert	17					1		1	19
Radius	3								3
Rib	37	6	3	4		1	3		54
Sacrum	2								2
Scapula	1			1		3	1		6
Sternum	2								2
Thoracic vert	21				1				22
Tibia	4								4
Ulna	2	1	1	1					5
Total	111	7	4	7	1	5	4	1	140

**Fig. 3:**
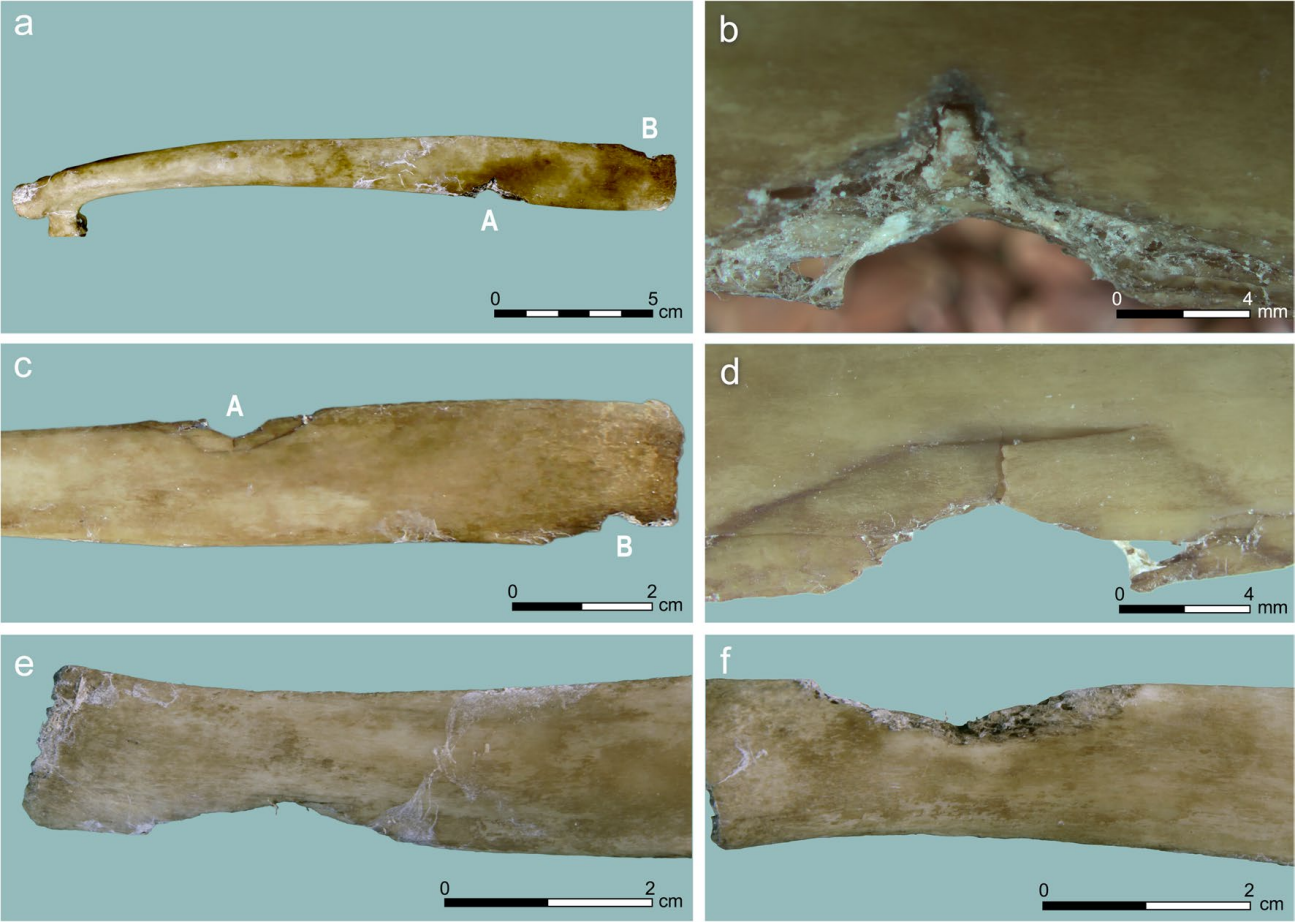
Notches PIM generated in our experiment: (**a**–**d**) a rib (#93) with two notches from two separate hits. Notch A shows entry in posterior position (**a**, **b**) and offset internally (**b**, **d**). Notch B is a narrower indentation with no visible beveling; (**e**, **f**) a notch on a rib (#90), showing a lateral entry wound (**e**) and internal beveling on the exit wound, on the mesial side (**f**)

Nine *notches* were readily visible, all on flat bone parts: ribs, the proximal ulna, processes of vertebrae, and the scapula neck. The breadth of the notches is variable, ca. 5–30 mm. Some present an offset at the direction of the hit or internal beveling in the exit side (Fig. 3). The shape of the notches was always semi-circular or semi-oval, similar to experiments with osseous (Letourneux and Pétillon 2008: Fig. 2) and wooden tips (Gaudzinski-Windheuser et al. 2018: Fig. 22), with none having a sharp appearance as seen in microlith-tipped projectiles (Yeshurun and Yaroshevich 2014: Fig. 3c). However, experiments with lithic-tipped projectiles also produced semi-oval notches that are similar to the ones described above, not just angular ones (Smith et al. 2020).

**Fig. 4:**
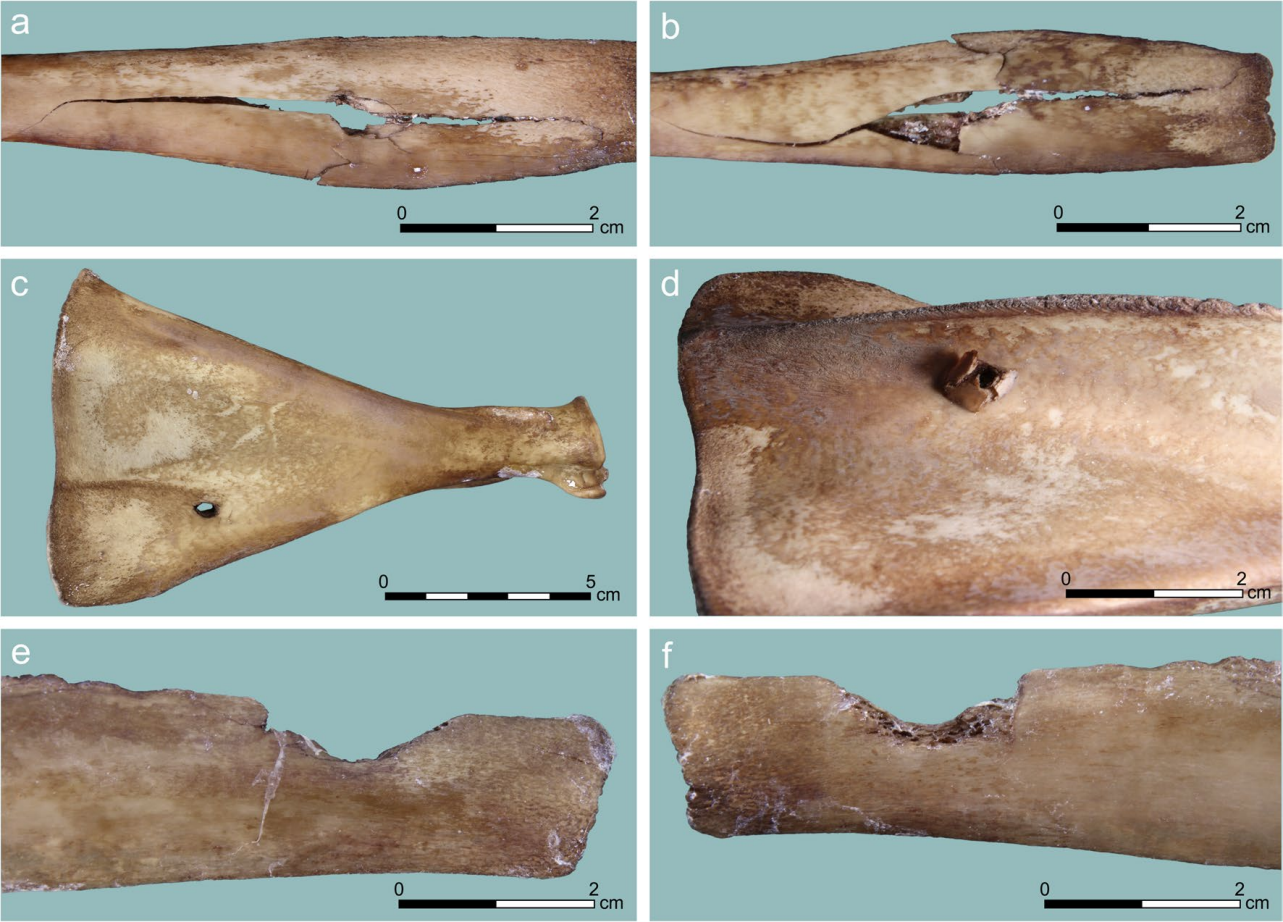
Perforation PIM generated in our experiment: (**a**, **b**) perforation and associated cracking on a rib (#5), entry (**a**) and exit (**b**) sides; (**c**, **d**) a small perforation on a scapula (#26), entry (**c**) and exit (**d**) sides, the latter with an offset; (**e**, **f**) a hemi-perforation (bordering a notch) on a rib (#83), showing the entry side (**e**) and internal beveling in the exit side (**f**)

Ten *perforations* occurred on scapula shoulder blades, ribs, and lumbar vertebrae (Table 2). Their appearance was very variable, even on the same skeletal element, from small oval holes that are ca. 5 mm in diameter, to irregularly shaped holes (often associated with cracking), to large oval holes that reach 20 mm in maximum diameter (Fig. 4). The exit wound and direction of the hit are conspicuous: internal beveling appeared in three cases (30% of perforations) and some offset in the direction of the hit appeared on other six specimens (60%). As with notches, the oval to round perforations generally differ from the more angular shape of lithic projectiles (Parsons and Badenhorst 2004: Fig. 1, 2), but resemble some of the perforations made experimentally by other osseous (Letourneux and Pétillon 2008: Fig. 4, 8) and wooden projectiles (Gaudzinski-Windheuser et al. 2018: Fig. 21). However, ours and the aforementioned experiments also produced more angular perforations, similar to the lithic-induced ones in Smith et al. (2020).

**Fig. 5:**
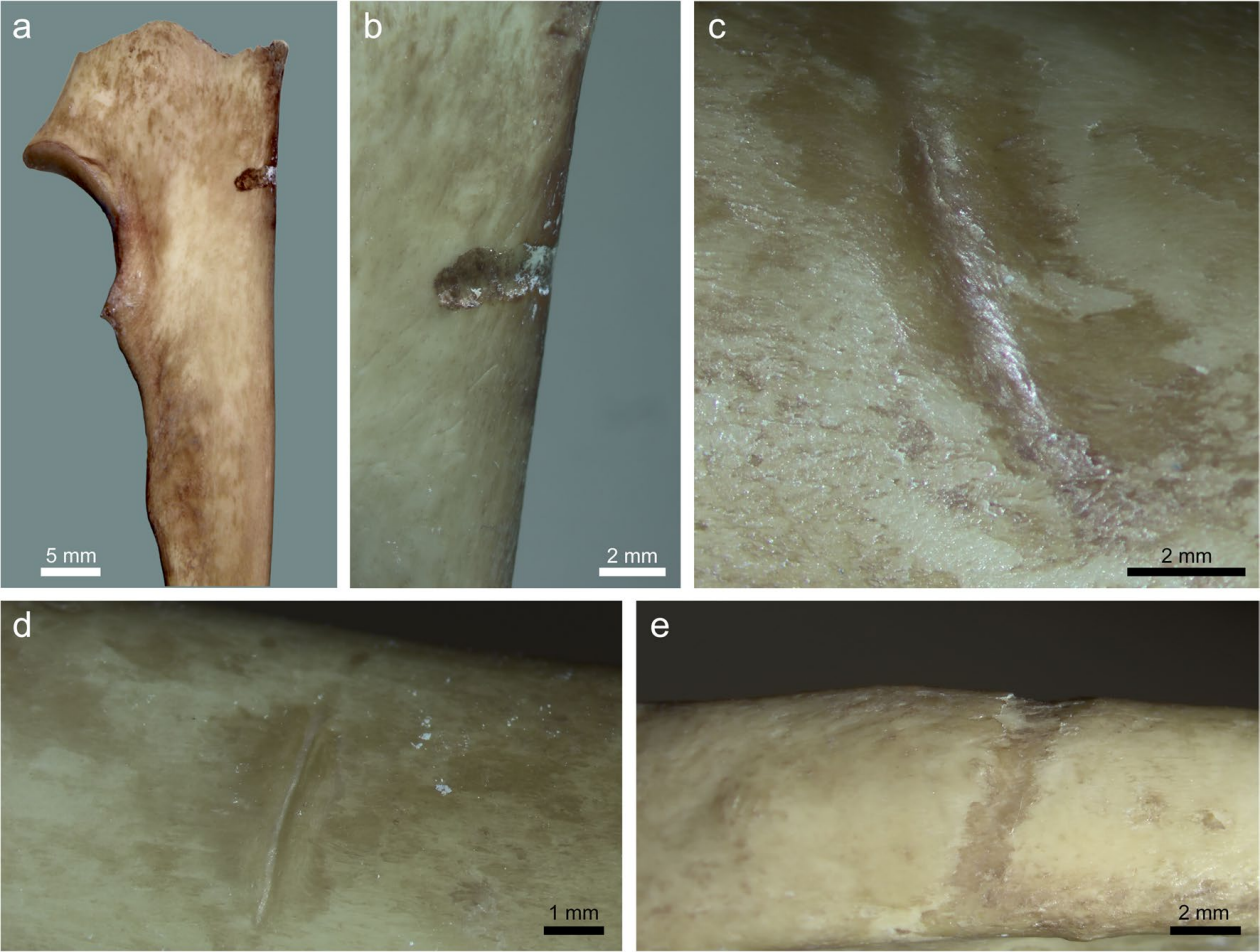
Striation PIM generated in our experiment: (**a**, **b**) a wide striation, perpendicularly oriented, on a proximal ulna (#64); (**c**) a narrower but still U-shaped cross-section striation on a rib shaft (#76). Note the abraded area around the striation; (**d**) a perpendicularly oriented striation on a rib (#81); (**e**) a wider lesion across the entire height of rib #81, with U-shaped cross-section

*Striations* were noted in four cases, on ribs and a proximal ulna (Table 2). They are oriented perpendicularly or obliquely to the long axis of the bones and exhibit a wide to narrow U-shaped cross-section, in one case with a slightly curved trajectory (Fig. 5). These four examples will probably not be confused with cutmarks because they lack the sharper, straight trajectory with a clear V-shaped cross-section. However, they bear resemblance to carnivore tooth scores in the generally straight, U-shaped and wide morphology. We did not observe clear micro-striations within the mark trajectory, in contrast with some lithic projectile experiments (O’Driscoll and Thompson 2014).

**Fig. 6:**
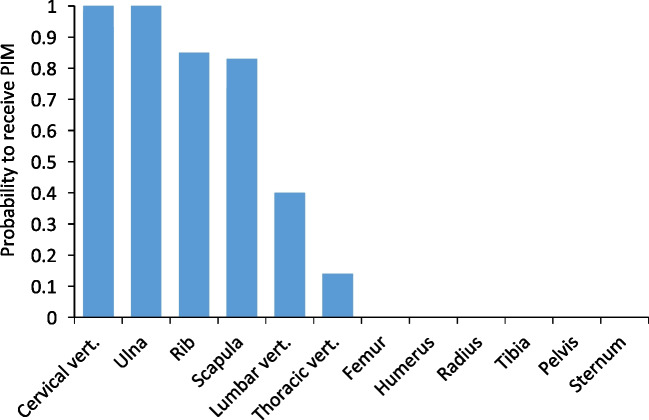
Comparison of the probability of different bones to receive impact damage (i.e., *N* of modified bones divided by number of documented hits at these bones). Data from Table 3

**Fig. 7:**
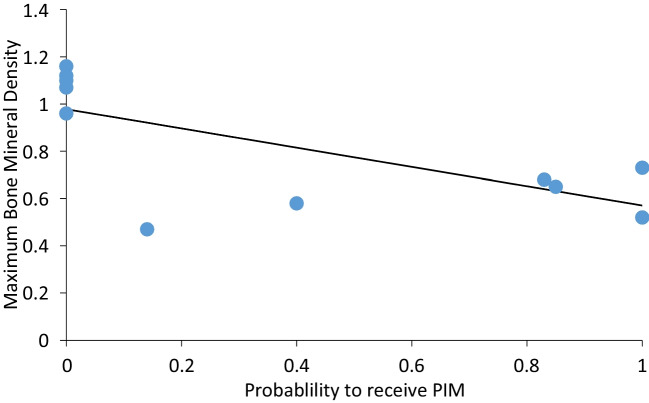
The relationship between the probability of a bone to receive impact damage, and its resilience to archaeological attrition process, approximated by the bone mineral density values (Lam et al. 1999: Table 1)

**Fig. 8:**
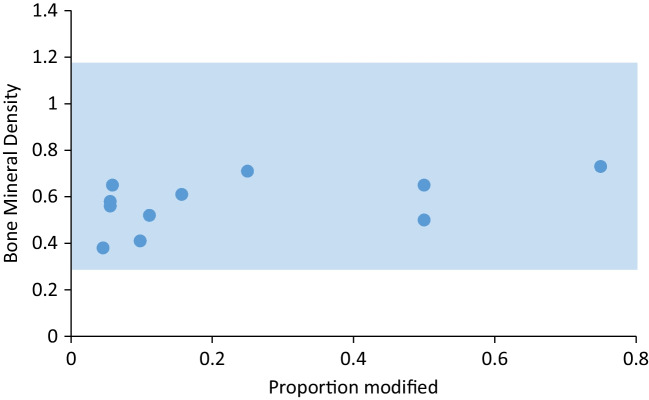
The relationship between the proportion of modified bone portions in our experiment and the preservation potential (bone mineral density values) of the same portions. The range of bone mineral density values is shaded

We found that the probability of a skeletal element to be modified by a hit (the number of PIM divided by the number of hits per bone) was very uneven (Fig. 6; Table 3). All hits to the cervical vertebrae and ulna, and most hits of the scapula and ribs, produced PIM of various types. The thoracic and lumbar vertebrae were affected as well, though by lower proportions. In contrast, the tubular limb bones were not marked by any hit. In other words, flat bones are overrepresented in our samples that yielded PIM. While our sample is small, it produced a clear trend (Fig. 7); the probability of a bone to be marked by PIM is inversely correlated with its preservation potential (approximated by the maximum bone mineral density values for each bone; Spearman’s *r* =  − 0.70, *p* = 0.01). Looking into the preservation potential of particular bone portions, all the PIM are manifested on skeletal elements in the lower range of the bone mineral density values, and therefore the lower preservation potential (Fig. 8).

**Table 3:** Counts of potential bone hits, as recorded during the experiment after each shot; the count of bones with PIM modification for each element; and the probability index, demonstrating how likely a bone is to exhibit PIM

	*Capreolus*	*Ovis*	Total	*N* modified	Index of sensitivity to shooting injury (*N* modified divided by *N* hit)
Cervical vertebra	1	0	1	1	1.00
Femur	2	0	2	0	0.00
Humerus	2	1	3	0	0.00
Lumbar vertebra	1	4	5	2	0.40
Pelvis	1	0	1	0	0.00
Radius	0	3	3	0	0.00
Rib	6	14	20	17	0.85
Scapula	4	2	6	5	0.83
Sternum	2	3	5	0	0.00
Thoracic vertebra	3	4	7	1	0.14
Tibia	0	1	1	0	0.00
Ulna	1	2	3	3	1.00

## Discussion

Bone modifications, and particularly PIM, are among the most straightforward proxies of activity and agency in archaeology. Specifically, PIM have been employed to link faunal resources to particular hunting weapons and tactics (e.g., Noe-Nygaard 1974; Bratlund 1991; Leduc 2014; Pöllath et al. 2018), and to assess the degree of human con- and inter-specific violence (e.g., Churchill et al. 2009; Crevecoeur et al. 2021). The results of our shooting experiment contribute to the more accurate description and identification of PIM in general and PIM from osseous-tipped weapons in particular. They also contribute to a better understanding of the PIM paucity problem in zooarchaeology. Three caveats about our data are, firstly, that we shot osseous-tipped projectile implements, and therefore not all patterns may be equally applicable to lithic tipped-projectiles; judging by the high concordance with previous experimental studies (see below), it seems that our data are mostly applicable for all kinds of projectiles. Second, our experimental design and discussion focuses on small-/medium-sized cervid/bovid. The different size and build of small mammals or the increased mass and tougher skins of large ungulates could change PIM abundances, types, and anatomical distribution. Third, the conclusions above are based on one experiment and, while they agree with many qualitative observations of previous experimental work, they still need to be substantiated with larger samples and additional, controlled variables.

While the identifiability and quantification of all bone-surface modifications are affected by preservation to some degree, PIM appear to be especially biased in this regard. Our experiment indicated that hitting an animal with a projectile actually has a good chance of producing bone modifications. Our aims were generally in line with those of hunters, as we intended to do maximal damage to the prey and not to cause damage on the bones. We mostly aimed at the center of the mass of the side of a small/medium ungulate hanging in real-life position. In spite of this, as much as half of our shots resulted in some bone damage. PIM appeared to be numerous in previous experiments as well, though they are usually not quantified relative to the number of potential hits (Castel 2008; Yeshurun and Yaroshevich 2014) or were derived from selectively shooting at specific body-parts or disarticulated carcasses (Smith et al. 2007; Badenhorst 2012; Gaudzinski-Windheuser et al. 2018).

The body size of the target animal appears to have an important effect on the abundance and location of PIM in the controlled experiments. Here, our experiment provided tentative data, in that the smaller-bodied target, the roe deer, was more susceptible to getting marked by PIM than the larger sheep (*Capreolus*: 14 PIM out of 23 potential bone hits, 61%; *Ovis*: 15 PIM out 34 potential bone hits, 44%). Other experiments suggested the same trend. Stodiek’s (1993) shots at a large ungulate, an old bison (*Bison bonasus*) and a medium-sized ungulate, fallow deer (*Dama dama*) produced no PIM on the former, but many on the latter. Shooting at a medium-sized ungulate, mouflon (*Ovis musimon*) produced PIM at 49% of the shots vs. 86% on the small mammal, coypu (*Myocastor coypus*) in Duches et al.’s (2020) experiments with microlith-tipped projectiles. Smith et al.’s (2020) experiments on wild boars (*Sus scrofa*) produced PIM on one-third of the bones, perhaps due to the stockier build of wild boar relative to the small/medium cervids and caprines. Thus, the inverse correlation between target body-size and PIM abundance seems clear, even if the inter-analyst results are not directly comparable due to different weapon types and velocities. However, in archaeological context, this clear trend might not be attested, due to the use of bigger projectiles, or different types, on bigger animals to ensure penetration and disabling of the hunted individual (Churchill 1993).

The high potential abundance of PIM on small/medium ungulates contradicts one of the explanations to the PIM paucity problem that proficient hunters would rarely hit bones. This paints an overly optimistic picture for taphonomists. Some expect the incidence of PIM to rise when this type of damage is systematically included in the research design and explicitly sought (Leduc 2014; O’Driscoll and Thompson 2018). This is surely the case, and we strongly support the inclusion of PIM into analyses of bone-surface modifications, but we expect this practice to yield a handful of, or none, PIM in the majority of Paleolithic faunal assemblages. This is due to two intertwined taphonomic problems that were quantified here.

First, many skeletal elements are unlikely to be marked when hit, and the probability of a PIM to occur is actually high in the skeletal elements that are the least likely to preserve intact due to their lower structural density. This was observed before in multiple experiments (e.g., Castel 2008; Badenhorst 2012) and is now statistically demonstrated by calculating the probability of a bone to obtain PIM in our experiment. The vertebrae, ribs, scapula blade, and the ulna olecranon were marked by all or most of the hits they took, while the long bones were not, despite being hit on several occasions. Due to their spongy structure, ribs and vertebrae are vulnerable to a range of pre- and post-discard processes and are preserved less often or in a less complete state than the most durable post-cranial elements such as the limb bone shafts.

An exception to this pattern was observed with young animals, whose bones are not completely ossified and exhibit lower structural density. Letourneux and Pétillon’s (2008) experiment included an adult fallow deer and a very young cattle calf. The adult deer produced very similar results to our experiment, where many shots were deflected off the long bones without damaging the bone. In contrast, the calf bones produced a higher number of marks and frequent punctures of the limb epiphyses. Our adult sheep and sub-adult roe deer targets compare well with their adult target (fallow deer) and also with the typical Paleolithic game, which is usually devoid of very young ungulate individuals.

The second taphonomic problem that heavily affects PIM presence and abundance is the non-diagnostic appearance of some PIM types. This observation was repeatedly stated by some experiments (e.g., Castel 2008; O’Driscoll and Thompson 2014; Duches et al. 2016, 2020). In this respect, the six PIM types differ markedly. The presence of the most unambiguous type of PIM, embedded lithic, is obviously dependent on the type of weapon used. We suspect that the rate of embedding in osseous projectiles would be consistently lower relative to lithic (or composite) ones. Our experiment produced no clear embedding of osseous tip fragments, whereas lithic embedding is normally present in experiments that employed lithics, and especially thin bladelets (Yeshurun and Yaroshevich 2014; Duches et al. 2020). Antler points rarely get embedded in bones during experimental shots (Stodiek 1993; Letourneux and Pétillon 2008). The higher resilience and longer use-life of organic projectile tips compared to lithics may lower the rate of “embedding” PIM in certain archaeological contexts. Osseous or wooden-tipped projectiles would rarely embed tip material in bone punctures and if they do, the material will not be preserved or identified.

Less clear-cut, but still rather distinctive, are perforations and notches on flat bones. When these exhibit directionality (offset) and internal beveling, one can recognize them as PIM, as other agents are unlikely to cause similar damage (Letourneux and Pétillon 2008; O’Driscoll and Thompson 2014; Yeshurun and Yaroshevich 2014; and the present study).

In contrast, punctures (incomplete perforations) can mimic carnivore tooth pits (Russo et al. 2023); the high variability in dimensions of carnivore- and projectile-induced pits complicates the differential diagnosis in many cases. As our experiment did not produce any punctures, we cannot contribute to this issue.

Cracking and striation types are especially challenging to diagnose. Cracking damage, which was especially evident on ribs and readily visible in our controlled experiment, would be invisible or nondiagnostic in all but the most exceptional archaeological circumstances. There is no way of telling if a rib broke due to cracking from a projectile hit or from butchery, trampling, or sediment compaction. Natural and biological fragmentation can lead to equifinality in interpreting crack damages. Another ambiguous, though less frequent, PIM type are linear striations; some bear a sharp appearance and could be confused with cutmarks (Smith et al. 2007), whereas the broader striations evident in our experiment could be confused with carnivore scores. Based on a large experimental sample, O’Driscoll and Thompson (2014) suggested criteria of diagnosis for striations (“drag marks”) that were further quantitatively assessed by Duches et al. (2016, 2020). Contextually, PIM striations are often associated with cracking, which is not the case for cutmarks and carnivore tooth scores. They often preserve signs of directionality, being the product of a single action with a clear trajectory, unlike butchery with stone tools that tend to be recurrent. The most significant morphological criteria were the breadth of striations, which is bigger than cutmarks but comparable to carnivore tooth scores, and their depth, which was larger than both types. Quantitative studies of modifications in specific archaeological contexts enable the diagnosis of the well-preserved linear striations (Duches et al. 2016, 2020), but these have to evaluated on a case-by-case basis, due to the large variability of PIM (in terms of velocity, weapon design, and hunting circumstances) and butchery marks.

The two analytical problems we quantified here obscure the occurrence of PIM, which may be ubiquitous (in terms of number of individual animals) and consistently present in the pre-burial stage. Unlike other anthropic marks resulting from butchery, hammerstone percussion, cooking, and bone-working, PIM are more likely to be present on one bone or only a few bones per individual, especially when the hunters are skilled, and are preferentially left on the bones that are the least likely to preserve and be present archaeologically. They also display a high rate of equifinality, leading to an artificially low rate of PIM identification. Many Paleolithic bone assemblages are biased against low-density elements in a ratio of 5 to 1; the initial abundance of PIM would thus decrease from ca. 50% of individuals (minimally, since an animal may be hunted with multiple shots) to ca. 10%. Then, in terms of identifiability, 37% of our PIM (eleven cases where cracking or striations were the sole mark) would not be diagnostic enough to be recognized as such, even if preserved. Thus, when PIM occur archaeologically, their presence should be taken as a minimum estimate, and their absence should not be interpreted as the absence or rarity of projectile technology (see discussion in Gaudzinski-Windheuser 2016 and Smith et al. 2020).

Some exceptionally preserved bone assemblages may approach the original abundance of PIM, for example, human or animal remains in primary interments, which were shielded from consumption, ravaging, weathering, and trampling damage. A case in point is the Late Paleolithic cemeteries of Nubia in the Nile Valley, where 41% of the individuals exhibit PIM, alongside other evidence of interpersonal violence (Crevecoeur et al. 2021); no PIM evidence presently exists in the faunal assemblages that were taphonomically analyzed from the same or contemporaneous contexts (Yeshurun 2018), despite the likelihood that the animals, too, were shot at by similar weapons. The possible loss of PIM from this faunal record is very likely to be the outcome of the combined effect of butchery, consumption, fragmentation, and erosion processes that food refuse had undergone, contrary to the interred human remains. When interred remains are properly examined and reveal an absence of PIM, this could be taken genuinely as absence of projectile weaponry use or interpersonal violence in that context.

Assuming that at least some PIM stand the chance of being preserved in the archaeological record, our results qualitatively contribute to the differential diagnosis that taphonomists need to consider when interpreting bone modifications. Our study supports other experimental data by demonstrating that it is sometimes possible to distinguish organic PIM from lithic ones. Since most studies, including ours, did not compare lithic and organic projectiles in the same experiments with all the other variables controlled for, these insights remain tentative. The notches and perforations obtained by shooting osseous-tipped weapons are round to oval in shape and almost never present angular edges (Stodiek 1993; Letourneux and Pétillon 2008). The notches and perforations created by thrusting or throwing a wooden spear include narrow and semi-circular notches as well as broader, semi-oval ones. Generally, they lack sharp and angular appearance and bear great resemblance to the notches generated in our experiment with osseous projectiles (Gaudzinski-Windheuser et al. 2018). These forms may be created by lithic weapons as well, but in the latter case the prevailing damage is that of angular lesions (O’Driscoll and Thompson 2014; Yeshurun and Yaroshevich 2014; Duches et al. 2016, 2020). Thus, PIM from organic tipped-weapons often result in the round or oval shape of perforations and semi-oval or semi-round notches on flat bones, in contrast to the generally sharp and angular break edges of lithic projectiles. Further separation between wooden and osseous PIM cannot presently be made by mark morphology alone.

## Conclusions

Our experimental data contributed a needed catalog of osseous-tipped PIM and corroborated the general features of bone injuries from organic weapon tips seen in previous studies. The most useful diagnostic features of any PIM in an archaeological assemblage are the notches and perforations, which bear directionality (seen by the offset) and internal beveling, both of which are unlikely to occur by other taphonomic agents. A round/oval shape of the perforations and notches would indicate organic-tipped weapons (wooden or osseous), rather than lithic-tipped ones. Additionally, we assessed the PIM paucity problem in zooarchaeology to suggest, albeit based on a single experiment, that PIM are potentially abundant in the pre-discard stage but significantly lost in the post-discard stage of typical bone assemblages generated by human hunting. PIM preferentially affect the structurally weakest skeletal elements, and some mark morphologies are not diagnostic enough in a complex taphonomic system to unequivocally link the traces to projectile impacts. In any case, PIM should be integrated in the research design and when found, it should probably be considered as a minimum estimate.

This experiment hopefully contributes to future studies that consider projectile hunting and their traces in the archaeofaunal assemblages from technological and zooarchaeological perspectives. Specifically, the results will serve to improve the association between osseous projectile tips and game remains in the Upper Paleolithic of Europe and the Levant.

### Supplementary Information

A catalog of the specimens with PIM from our experiment: 3D pdf files scanned using Arctec Spider. Each specimen file is identified by its catalog number and type/s of PIM (see Table 1).

Supplementary file1 (PDF 25923 KB) Yeshurun_etal_123_perforations_notch

Supplementary file2 (PDF 45181 KB) Yeshurun_etal_136_notch

Supplementary file3 (PDF 4807 KB) Yeshurun_etal_13_cracking

Supplementary file4 (PDF 4678 KB) Yeshurun_etal_15_cracking

Supplementary file5 (PDF 2700 KB) Yeshurun_etal_22_cracking

Supplementary file6 (PDF 4045 KB) Yeshurun_etal_24_cracking

Supplementary file7 (PDF 27759 KB) Yeshurun_etal_25_perforations_cracking

Supplementary file8 (PDF 27834 KB) Yeshurun_etal_26_perforation

Supplementary file9 (PDF 8220 KB) Yeshurun_etal_3-cracking

Supplementary file10 (PDF 13189 KB) Yeshurun_etal_38_notch

Supplementary file11 (PDF 5235 KB) Yeshurun_etal_45_cracking

Supplementary file12 (PDF 7637 KB) Yeshurun_etal_5-perforation_cracking

Supplementary file13 (PDF 2843 KB) Yeshurun_etal_51_cracking

Supplementary file14 (PDF 4223 KB) Yeshurun_etal_51_notch

Supplementary file15 (PDF 5470 KB) Yeshurun_etal_55_cracking

Supplementary file16 (PDF 3339 KB) Yeshurun_etal_56_perforation_cracking

Supplementary file17 (PDF 4761 KB) Yeshurun_etal_64_cracking_striation

Supplementary file18 (PDF 12611 KB) Yeshurun_etal_76_striation

Supplementary file19 (PDF 16981 KB) Yeshurun_etal_81_striations

Supplementary file20 (PDF 23572 KB) Yeshurun_etal_83_perforation_notch

Supplementary file21 (PDF 23809 KB) Yeshurun_etal_90_notch

Supplementary file22 (PDF 4696 KB) Yeshurun_etal_92-1_cracking

Supplementary file23 (PDF 17727 KB) Yeshurun_etal_92-2_cracking

Supplementary file24 (PDF 18159 KB) Yeshurun_etal_93_notches

Supplementary file25 (PDF 18219 KB) Yeshurun_etal_96_perforation

Supplementary file26 (PDF 82408 KB) Yeshurun_etal_98_perforation_notch1

Supplementary file27 (PDF 6098 KB) Yeshurun_etal_98_snapped part
